# First record of the complete mitochondrial genome of *Tubifex tubifex* (Müller) 1774 (Annelida; Clitellata; Oligochaeta) and phylogenetic analysis

**DOI:** 10.1080/23802359.2022.2032856

**Published:** 2022-07-04

**Authors:** Jeounghee Lee, Jongwoo Jung

**Affiliations:** a Marine Biological Resource Institute, Sahmyook University, Seoul, Republic of Korea; b Department of Science Education, Ewha Womans University, Seoul, Republic of Korea

**Keywords:** Freshwater Oligochaeta, *Tubifex tubifex*, complete mitochondrial genome, phylogenetic analysis

## Abstract

The complete mitochondrial genome of *Tubifex tubifex* was analyzed using the MGISEQ-2000 platform. The size of the complete mitochondrial genome was 15,972 bp. Data pertaining to the genome, such as the presence of 13 protein-coding genes (PCGs), two rRNA genes, 22 tRNA genes, and a putative control region were submitted to NCBI (MW690579). A phylogenetic tree was constructed with the sequences of the 13 PCGs using the maximum-likelihood method. Despite only a few references available on the complete mitochondrial genome of other aquatic oligochaetes, our phylogenetic analysis revealed that the freshwater oligochaetes *T. tubifex* and *Limnodrilus hoffmeisteri* are in a cluster different from that of the earthworm group.

*Tubifex tubifex* belongs to the subfamily Tubificinae of Oligochaeta. It inhabits mud and/or sewage sludge in freshwater habitats and can survive in environments with polluted water. *T. tubifex* is a bioindicator of environmental conditions (Kerans et al. 2009; Kaonga et al. 2010; Spica et al. 2014). *T. tubifex* is found worldwide; however, molecular studies are limited, compared to morphological and environmental studies. To date, the mitochondrial genome of freshwater oligochaetes has not been studied (Anlauf and Neumann 1997; Beauchamp et al. 2001; Achurra et al. 2011). Therefore, in this study, the complete mitochondrial genome of *T. tubifex* was assembled and compared to that of other earthworm species. This result could provide useful information about the genetics and evolutionary processes of *T. tubifex* and other aquatic oligochaetes.

Specimens were collected from Seoul (Korea) in June 2019 (127° 04′ 41; 74″E 37° 34′ 52.87″N) and preserved in 80% ethanol. A voucher specimen was deposited at the National Institute of Biological Resources (https://www.nibr.go.kr, Hyun Ki Choi and choi3112@korea.kr) under the voucher number KDELIV0000003033. Whole genomic DNA was extracted from the posterior body segments of an adult specimen using a REPLI-g Mitochondrial DNA Kit (Qiagen, Germantown, MD). Whole-genome sequencing was performed using the MGISEQ-2000 platform. The mitochondrial genome was constructed using MITObim v1.9.1 (Hahn et al. 2013) and MITOS (Bernt et al. 2013). Annotations were generated using Geneious Prime 2019.2.1 (Kearse et al. 2012). Alignment of genome data from *T. tubifex*, eight oligochaete species, one leech species, and one polychaete species (outgroups) was performed using Clustal W (Thompson et al. 2003). The phylogenetic tree was constructed based on the sequences of 13 protein-coding genes (PCGs) using the maximum-likelihood (ML) method with IQ-TREE (Nguyen et al. 2015). The GTR + G+I model was identified as the best-fit model for the data, using ModelFinder (Kalyaanamoorthy et al. 2017) with 1000 bootstrap replicates.

The size of the complete mitochondrial genome was 15,972 bp, and the data were submitted to the NCBI (MW690579). The genome consisted of 62.7% A + T bias (A = 31.2%, C = 22.3%, G = 15.0%, and T = 31.5%). In addition, it included 13 PCGs, two rRNA genes, 22 tRNA genes, and a putative control region consisting of 678 bp. The PCGs used diverse start codons, including ATG (*ATP8, COX1, COX2, COX3, CYTB, NAD1, NAD2, NAD4, NAD4L,* and *NAD5*), ATT (*ATP6* and *NAD6*), and ATC (*NAD3*). The phylogenetic relationship of *T. tubifex* with the other members of subclass Oligochaeta was assessed using the ML method (Figure 1). Our phylogenetic analysis revealed that *T. tubifex* is clustered with *Limnodrilus hoffmeisteri* and *Nais communis* with a high support value (100/95), indicating that freshwater oligochaetes are in a location different from the earthworm group.

**Figure 1.**
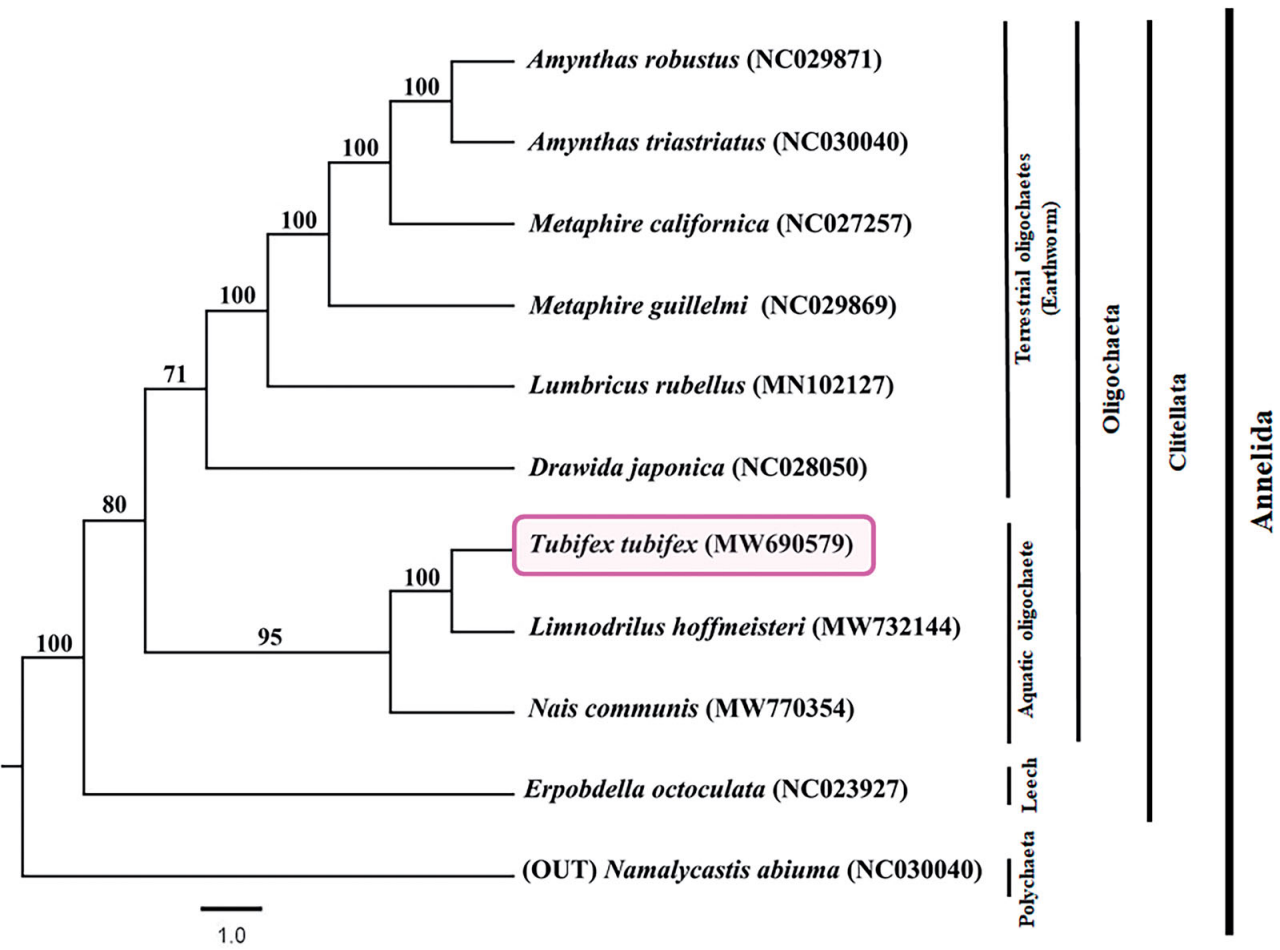
Molecular phylogeny of *Tubifex tubifex* (MW690579), a species of freshwater oligochaete, eight species of annelids, and an outgroup species based on the nucleotide sequences of 13 protein-coding genes (PCGs). The complete mitogenomes are downloaded from GenBank and the phylogenetic tree is constructed by the maximum-likelihood (ML) method with 1000 bootstrap replicates.

Taken together, these results can help assign the phylogenetic position of aquatic oligochaetes in the Annelida phylum.

## Data Availability

The genome sequence data that support the findings of this study are openly available in GenBank (National Center for Biotechnology Information) at https://www.ncbi.nlm.nih.gov, accession no. MW690579. The associated BioProject, SRA, and Bio Sample numbers are PRJNA725065, SRR14447867, and SAMN18869252, respectively. The data that support the findings of this study are also openly available in Mendeley Data at http://dx.doi.org/10.17632/c45jzr8ksx.2
